# Effectiveness of COVID-19 Vaccines against SARS-CoV-2 Omicron Variant (B.1.1.529): A Systematic Review with Meta-Analysis and Meta-Regression

**DOI:** 10.3390/vaccines10122180

**Published:** 2022-12-19

**Authors:** Nando Reza Pratama, Ifan Ali Wafa, David Setyo Budi, Henry Sutanto, Tri Pudy Asmarawati, Gema Barlian Effendi, Citrawati Dyah Kencono Wungu

**Affiliations:** 1 Faculty of Medicine, Universitas Airlangga, Surabaya 60115, Indonesia; 2 Department of Cardiology, CARIM School for Cardiovascular Diseases, Maastricht University, 6211 Maastricht, The Netherlands; 3 Department of Internal Medicine, Universitas Airlangga Hospital, Universitas Airlangga, Surabaya 60115, Indonesia; 4 Institute of Tropical Disease, Universitas Airlangga, Surabaya 60115, Indonesia; 5 Department of Physiology and Medical Biochemistry, Faculty of Medicine, Universitas Airlangga, Surabaya 60115, Indonesia

**Keywords:** COVID-19, SARS-CoV-2, Omicron variant, vaccine effectiveness, booster vaccination

## Abstract

Vaccine effectiveness (VE) and the urgency of booster vaccination against SARS-CoV-2 Omicron variant need evaluation. A systematic search was conducted from 1–6 April, 2022. VE difference (VED) estimates were assessed using random-effects and meta-regression analyses were performed for evaluating VE over time. Compared to full dose, booster dose of overall vaccines provided better protection against any and severe Omicron infections within 3 months (*p* < 0.001), and within 3 months or more in any, severe, and symptomatic infections (*p* < 0.001). From meta-regression analysis of overall vaccines, the full-dose VE against any and symptomatic Omicron infections reduced per month by 2.45% and 5.5%, respectively; whereas booster dose effectiveness against any and symptomatic Omicron infections reduced per month by 1.79% and 1.14%, respectively. The VE estimates of booster dose provide excellent protection against symptomatic infection compared to full dose. The VE estimates of Ad26.COV2.S, BNT162b2, ChAdOx1 nCov-19, and mRNA-1273 against Omicron infection are generally moderate, despite the VE estimates declining over time.

## 1. Introduction

As of April 2022, there were at least two circulating SARS-CoV-2 variants of concern: the B.1.617.2 (Delta) and B.1.1.529 (Omicron) variants [1]. In November 2021, the Omicron variant was first identified in South Africa and was immediately declared a variant of concern by the World Health Organization. Alongside the massive rise in the confirmed cases of SARS-CoV-2 infection in South Africa, the Omicron variant started to spread across the globe in no time. The identification of several concerning mutations in the SARS-CoV-2 Omicron variant and evidence of an enhanced immune escape ability contributed to the rapid spread of the Omicron variant worldwide [2]. Compared to the ancestral variants (Wuhan-Hu-1 or Wuhan-1), the Omicron variant contains more mutations (i.e., 60 mutations), 32 of which occur in the spike gene which encodes the primary antigen target for a wide variety of COVID-19 vaccines [3]. These mutations have been linked to increased transmissibility, a high rate of immune evasion following natural infection and vaccination, and the impairment of the efficacy of SARS-CoV-2 vaccines [4].

Vaccine effectiveness (VE) is a measure of how well vaccines protect people from infections in the real-world setting [5]. It played a critical role in restricting the spreading of SARS-CoV-2 infections in the current COVID-19 pandemic [6]. Earlier in the COVID-19 pandemic, studies predicted a 60–90% herd immunity threshold to limit the disease spreading, which could be achieved through several measures, including a mass vaccination campaign [7,8]. Indeed, several COVID-19 vaccines have been shown to be promising by numerous large randomized-controlled trials (RCTs) [9,10,11,12,13]. Since then, many countries have extensively implemented COVID-19 vaccination programs. However, prior laboratory and clinical studies have indicated a reduction in VE against the Omicron variant as compared to the earlier variants [14,15,16], potentially affecting the current COVID-19 vaccination strategy. Therefore, with the surge of new SARS-CoV-2 variants, booster vaccine doses were administered to confer stronger immunity, which hopefully could increase VE [17,18]. However, due to global disparity in the availability and distribution of COVID-19 vaccines and the vaccination rates, developing countries were pushed to expedite booster vaccination with (limited) available resources to foster their booster vaccination rates [19,20].

Since an equal distribution of booster vaccines remains a challenge and Omicron’s spike antigen landscape is heavily altered, there is a need for explorations regarding the effectivity of currently available vaccines and the urgency of booster vaccination against the SARS-CoV-2 Omicron variant. Here, we performed systematic review and meta-analysis to unravel the effectiveness of full and booster vaccinations against the SARS-CoV-2 Omicron variant.

## 2. Materials and Methods

This systematic review conformed with the guidelines of Preferred Reporting Items for Systematic Review and Meta-Analysis (PRISMA) 2020 [21] and has been registered in the PROSPERO database (CRD42022302267).

### 2.1. Eligibility Criteria

This review included any study designs, including RCT, cohort, case-control, and cross-sectional studies. Studies were selected according to the following criteria: (1) administration of COVID-19 vaccine during the Omicron variant’s wave as the study of interest; (2) eligible studies reporting at least one of our outcomes of interest; and (3) English language. Our outcomes included VE difference (VED) between the booster and full dose, the correlation of booster dose VE with time, and the correlation of full-dose VE with time. We excluded review articles, nonhuman studies, irrelevant articles, and duplications.

### 2.2. Search Strategy and Selection of Studies

**Figure 1:**
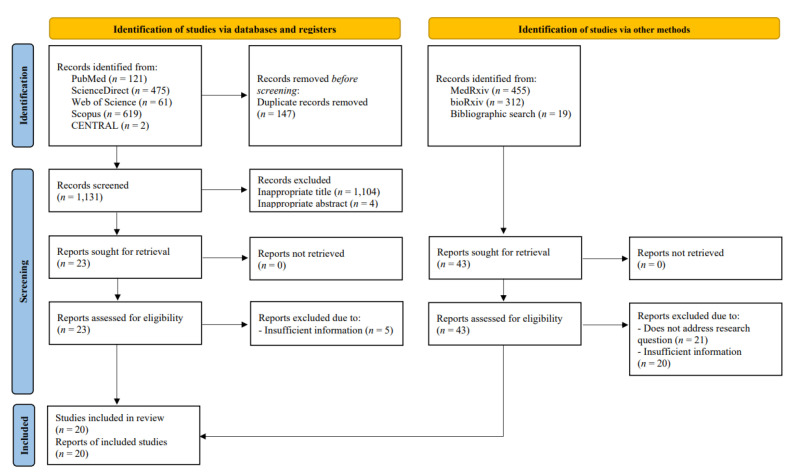
PRISMA flow diagram of study selection process.

Two authors (I.A.W and D.S.B) conducted a keyword search for articles published in databases (PubMed, ScienceDirect, Cochrane Central Register of Controlled Trials [CENTRAL], Web of Science, and Scopus) from 1 to 6 April 2022. Extended manual search (e.g., in medRxiv, bioRxiv) and bibliographical search were also conducted to obtain additional potential articles. The following keywords were used: “((SARS-CoV-2) OR (COVID-19)) AND ((Omicron) OR (B.1.1.529)) AND ((Vaccine) OR (Vaccination)) AND ((Vaccine efficacy) OR (Vaccine effectiveness))”. Detailed search strategies are available in Supplementary Materials (Table S1). We exported all studies retrieved from the electronic search into the Mendeley reference manager for duplication removal and independent screening. Any disagreements between these two authors were resolved by discussion with all authors until consensus was reached. The number of excluded studies was specified in the PRISMA flow diagram alongside their reasons for exclusion (Figure 1).

### 2.3. Data Extraction

Two review authors (N.R.P. and D.S.B.) independently extracted relevant data from each selected study using a structured and standardized form. For each included study, the following relevant data were collected: first authors’ names and publication year, study design, country of origin, sample size, patient age, Omicron strain confirmation method, follow-up duration, dose, types and administration interval of COVID-19 vaccines, endpoints, and VE.

### 2.4. Quality Assessment

The methodological quality of each study was assessed independently by two authors (I.A.W and D.S.B) using the original Newcastle–Ottawa Scale (NOS) for case-control and cohort studies [22]. The tool evaluates the quality of observational studies from the following 3 domains: (1) sample selection; (2) study comparability; and (3) study outcome. The NOS contains 8 items with scores ranging from 0 to 9. The total score of 0–3, 4–6, and 7–9 indicated low-, moderate-, and good-quality studies, respectively. Any discrepancies were resolved by discussion until consensus was reached.

### 2.5. Outcomes Measure

We evaluated three outcomes: (1) VED between the booster and full dose, which was evaluated by two models: ‘within 3 months’ and ‘within 3 months or more’; (2) correlation of booster dose VE with time; and (3) correlation of full-dose VE with time. We further evaluated each of these outcomes for three different endpoints: any, symptomatic, and severe Omicron infections. The ‘any infection’ endpoint included positively-tested COVID-19, symptomatic COVID-19, and severe COVID-19. Meanwhile, the ‘symptomatic infection’ endpoint comprised any individuals who had tested positive and showed COVID-19 symptoms as well as individuals who required hospital visits without hospitalization. Those who were hospitalized due to COVID-19, regardless of the received treatment, were deemed as having a severe infection.

For VED between the full and booster doses, we analyzed the results using 2 models: the ‘within 3 months’ and the ‘within 3 months or more’ models. The ‘within 3 months’ model included data in the first 3 months reported by each study and the ‘within 3 months or more’ model included data in the first 3 months or more reported by each study.

### 2.6. Statistical Analysis

Primary analyses were carried out using R version 4.0.5 with meta and dmetar package. VE was calculated as (1 − OR) ×100% for case-control studies, while (1 − RR) × 100% or (1 − HR) × 100% for cohort studies. VED was defined as the difference of VE between the booster and full-dose vaccines, i.e., VE of booster dose—VE of full dose. We used the *I^2^* test to quantify the heterogeneity between studies, with values *I^2^* > 50% representing moderate-to-high heterogeneity. Random effects were used with the inverse variance method for pooling the results and DerSimonian–Laird for estimating τ^2^. Egger’s test was performed for the evaluation of publication bias. All statistical analyses with a *p*-value < 0.05 was considered statistically significant. Leave-one-out sensitivity analysis was conducted to find the source of statistical heterogeneity and demonstrate how each study influenced the overall result. Meta-regression analysis was also performed using inverse-variance and restricted-maximum likelihood with Hartung–Knapp adjustment. VE reduction per month was approximated by multiplying the VE reduction per day—the slope of VE vs. time—by 30.

## 3. Results

### 3.1. Study Selection and Quality Assessment

From databases and manual search, 1278 and 786 records were retrieved, respectively. A total of 147 duplicates were subsequently removed. Following the screening of titles and abstracts, 66 potential articles were selected for review. After a full-text review, 20 observational studies, consisting of 4 cohorts and 16 test-negative case-control studies, were included in the systematic review, meta-analysis, and meta-regression. The overall screening process of this systematic review and meta-analysis is summarized in the PRISMA flow diagram (Figure 1). The quality assessment of each study using the NOS critical appraisal checklist is listed in Tables S2 and S3. All included studies were considered good-quality studies according to the quality assessment.

### 3.2. Study Characteristics

We included 20 studies, consisting of 4 cohorts and 16 test-negative case-control studies with a total of 12,409,084 participants. The summary of study characteristics was tabulated in Table 1. Each study is further divided by the type of COVID-19 vaccines and the number of doses, i.e., full doses (Table S4) or booster doses (Table S5). Four types of COVID-19 vaccine (i.e., Ad26.COV2.S, BNT162b2, ChAdOx1 nCov-19, and mRNA-1273) were included in the VE analyses of the full and booster doses. Full-dose vaccination represents two doses of BNT162b2, ChAdOx1 nCov-19 and mRNA-1273 vaccines, or one dose of the Ad26.COV2.S vaccine, whereas booster dose vaccination was defined as the administration of an extra dose of COVID-19 vaccine on top of the full-dose vaccination. 

Six studies [29,31,32,34,35,37] used mRNA vaccines but did not further specify the manufacturers. In this case, we defined them as any mRNA vaccines. Two studies [38,42] evaluated the effectiveness of COVID-19 vaccines only among children and adolescents, while the rest of the studies included adults as their participants. Geographically, the included studies originated from seven countries/locations: twelve studies were conducted in the United States, three studies in Europe, two studies in South Africa, three studies in Qatar, and one study in Canada. The follow-up time intervals varied among studies. Between booster and full doses, only three studies [32,39,41] had a similar median or range from the date of dose receipt to the date of endpoint events. The VE was calculated as (1 − OR) × 100% among all case-control studies, while VE among cohort studies was calculated as (1 − OR) × 100%, (1 − RR) × 100%, or (1 − HR) × 100% (Table 1). The VE from each study with its 95% clinical interval (CI) for full and booster doses are summarized in Tables S5 and S6, respectively. The results of the VED calculation are summarized in Table S7.

### 3.3. VED Estimates between Booster and Full Dose

#### 3.3.1. Overall Analysis

**Figure 2:**
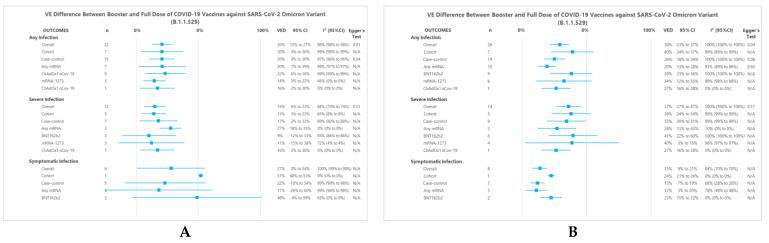
Forest Plot summary representing VED between the booster and a full dose of COVID-19 vaccine against SARS-CoV-2 infections. Panel (**A**) and (**B**) show subgroup summary of VED ‘within 3 months’ and ‘within 3 months or more’ models, respectively.

Results from two meta-analysis models, i.e., the ‘within 3 months’ (Figure 2A) and ‘within 3 months or more’ (Figure 2B) models, were compared. In the ‘within 3 months’ model, there were 22 separate analysis data involving any mRNA vaccines (*n* = 7), BNT162b2 (*n* = 9), mRNA-1273 (*n* = 5), and ChAdOx1 nCov-19 (*n* = 1). Meanwhile, in the ‘within 3 months or more’ model, we obtained 26 separate analysis data that involved any mRNA (*n* = 10), BNT162b2 (*n* = 9), mRNA-1273 (*n* = 6), and ChAdOx1 nCov-19 (*n* = 1). As a comparison between these two models, pooled results of the ‘within 3 months or more’ model generally had a higher VED, both for any infection or severe infection.

In the ‘within 3 months’ model, the pooled results for preventing any, severe, and symptomatic infection showed that booster dose had a significantly higher VE than that of the full dose (VED of 20% (95%CI 12% to 27%), 14% (95%CI 6% to 23%), and 27% (95%CI 0% to 54%), respectively). In the ‘within 3 months or more’ model, the pooled results also showed a better VE on booster dose for any, severe, and symptomatic infection (VED of 30% (95%CI 23% to 37%), 37% (95%CI 27% to 47%), and 15% (95%CI 9% to 21%), respectively).

Leave-one-out sensitivity analyses were performed on the overall analysis, yielding similar results in terms of effect estimates or statistical heterogeneity (Figures S1–S6). For publication bias analysis, Egger’s tests were significant only for the case-control subgroup of any infection at ‘within 3 months’ model (*p* = 0.04) (Figure 2A) and the overall analysis of ‘within 3 months or more’ model (*p* = 0.04) (Figure 2B). Egger’s test was not performed for subgroups with n < 10.

#### 3.3.2. Subgroup Analysis of ‘Within 3 Months’ Model

For any infection, the cohort and the case-control subgroup had a VED of 20% (95%CI 3% to 36%) and 20% (95%CI 9% to 30%), respectively. A further subgroup analysis with respect to vaccine type showed that any mRNA, BNT162b2, mRNA-1273, and ChAdOx1 nCov-19 had VEDs of 20% (95%CI 1% to 39%), 22% (95%CI 6% to 38%), 14% (95%CI 5% to 22%), and 16% (95%CI 2% to 30%), respectively. All of these results were statistically significant.

For severe infection, cohort and case-control subgroups showed significant results (VEDs of 13% (95%CI 3% to 23%) and 17% (95%CI 2% to 32%), respectively). A further subgroup analysis showed statistically significant results for VED of any mRNA and ChAdOx1 nCov-19 (VED 27% (95%CI 18% to 35%) and 16% (95%CI 2% to 30%), respectively).

For symptomatic infection, cohort and case-control subgroups also showed VEDs of 51% (95%CI 48% to 53%) and 22% (95%CI −10% to 54%), respectively. In any mRNA and BNT162b2 subgroups, the booster dose had a better VE than that of the full dose (VED of 17% (95%CI −26% to 60%) and 48% (95%CI −4% to 99%), respectively), yet these results did not reach statistical significance.

#### 3.3.3. Subgroup Analysis of ‘Within 3 Months or More’ Model

For any infection, the cohort subgroup had a VED of 40% (95%CI 24% to 57%), but results obtained from the case-control subgroup showed a lower VED, with a VED of 26% (95%CI 18% to 34%). A further subgroup analysis with respect to vaccine type showed that any mRNA, BNT162b2, mRNA-1273, and ChAdOx1 nCov-19 had VEDs of 20% (95%CI 13% to 28%), 39% (95%CI 23% to 56%), 34% (95%CI 12% to 55%), and 27% (95%CI 16% to 38%), respectively. All of these results were statistically significant.

For severe infection, cohort and case-control subgroups showed significant results (VEDs of 39% (95%CI 24% to 54%) and 35% (95%CI 20% to 51%), respectively). A further subgroup analysis showed that the VED of any mRNA, BNT162b2, mRNA-1273, and ChAdOx1 nCov-19 vaccine was 28% (95%CI 13% to 43%), 41% (95%CI 22% to 60%), 40% (95%CI 3% to 76%), and 27% (95%CI 16% to 38%) respectively. Likewise, all these results were statistically significant.

For symptomatic infection, cohort and case-control subgroups also showed significant results with VEDs of 24% (95%CI 21% to 26%) and 13% (95%CI 7% to 19%), respectively. In any mRNA and BNT162b2 vaccines subgroups, the booster dose also presented a better VE than that of the full dose (VEDs of 12% (95%CI 3% to 20%) and 23% (95%CI 15% to 32%), respectively). All of these results were statistically significant.

### 3.4. VE Estimates and VE Reduction for Booster Dose and Full Dose

**Figure 3:**
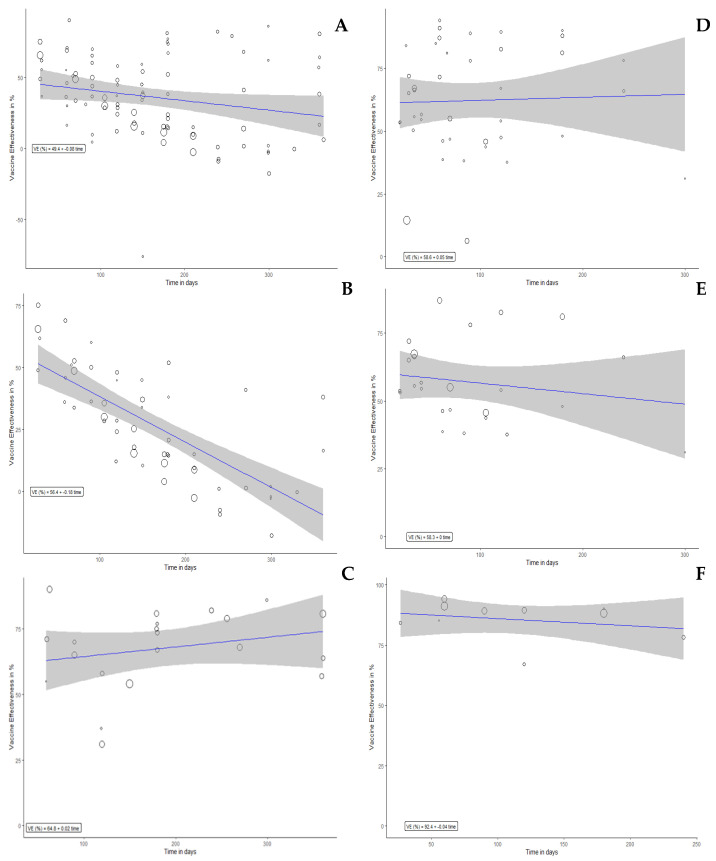
Meta-regression plot for VE vs. Time in days. VE on panels (**A**–**C**) represent VE of full dose against any infection, symptomatic infection, and severe infection, respectively. VE on panels (**D**–**F**) represent VE of booster dose against any infection, symptomatic infection, and severe infection, respectively. VE estimate (%) was 49.45 (95%CI 38.01 to 38.01), *p* < 0.001 (**A**); 56.36 (95%CI 47.21 to 47.21), *p* < 0.001 (**B**); 64.81 (95%CI 49.90 to 49.90), *p* < 0.001 (**C**); 56.78 (95%CI 47.11 to 66.45), *p* < 0.001 (**D**); 53.58 (95%CI 44.18 to 62.98), *p* < 0.001 (**E**); 92.53 (95%CI 85.54 to 99.52), *p* < 0.001 (**F**).

In the meta-regression analysis, the VE of full vaccination dose against any (Figure 3A), severe (Figure 3B), and symptomatic (Figure 3C) infections were significantly correlated with time (*p* < 0.001). The VE of booster vaccination dose against any (Figure 3D), severe (Figure 3E) and symptomatic (Figure 3F) infections were significantly correlated with time (*p* < 0.001). The correlation of VE (%) with time (day) was 49.45–0.08 per day (Figure 3A); 56.36–0.18 per day (Figure 3B); 64.81–0.02 per day (Figure 3C); 56.78–0.06 per day (Figure 3D); 53.58–0.04 per day (Figure 3E); 92.53–0.04 per day (Figure 3F).

For the full-dose vaccination, our meta-regression model estimated that the VE of full dose against any and symptomatic infections were decreased in each month approximately by 2.45% (95%CI 0.63% to 4.26%) and 5.5% (95%CI 3.99% to 7.01%), respectively. The detailed results displaying the correlation of VE with time for each vaccine were presented in Table 2.

## 4. Discussion

This study aimed to evaluate full-dose and booster VE and their correlation with time to evaluate waning immunity. We used two models for VED evaluation based on time period, i.e., ‘within 3 months’ and ‘within 3 months or more’. Our models demonstrated that there was a significant VED between the booster and the full dose in terms of preventing any, symptomatic, and severe infections of the SARS-CoV-2 Omicron variant. Although these results had a generally high heterogeneity, subgroup analyses showed that study design and types of vaccine did not seem to contribute to this heterogeneity. However, VED at a more prolonged interval model, i.e., ‘within 3 months or more’, was more elevated. Most analyses at a longer interval contained more full-dose vaccine data since the follow-up interval for the full vaccination dose was considerably longer than that of the booster dose.

On the other hand, our meta-regression analysis showed that the full-dose VE against any and symptomatic infections were estimated to be reduced by 2.45% and 5.5% each month, respectively. Meanwhile, for the booster dose VE reduction against any, symptomatic, and severe infections were insignificant. Additionally, VE estimates of booster dose were generally higher than those of full dose, in line with VED meta-analysis results. VE estimates of booster doses were generally at more than 50% for all endpoints. Booster doses of mRNA vaccines showed excellent protection against severe infection, with a VE of 94.54%, compared to full dose with a VE of 64.81%. This result was in agreement with the prediction by Khoury et al. [43] In that study, it was predicted that a booster dose could raise VE from 81.1% to 98.2% for mRNA vaccines. Moreover, in that study, BNT162b2 and mRNA-1273 were predicted to have more than 80% VE against severe infection, and VE against severe infection was generally higher than that of symptomatic infection [43].

Vaccination or natural infection induces some immune cell subsets to turn into memory cells through clonal expansions [44]. These previously primed cells could deliver a more robust immune response in the secondary response, which is protective against severe disease. Meanwhile, neutralizing antibodies can provide sterilizing immunity to prevent infection [44,45,46]. Neutralizing antibodies produced by plasma B cells decay over time, but long-lived plasma B memory cells continuously secrete neutralizing antibodies even after the infection ends, maintaining their level [47]. Moreover, a robust immune response and multiple infections or vaccinations can elicit strong immunoglobulin G (IgG)-binding affinity as a result of an affinity maturation process [48,49]. Consequently, compared to full vaccination doses, an additional booster vaccination dose would elicit a stronger immune response [50], as evidenced by some studies that reported higher binding affinity and titers of neutralizing antibodies among individuals who received three vaccination doses than that of two doses [50,51,52]. For instance, with regard to the Omicron variant, the antibody titer induced by a booster dose of BNT162b2 at 1 month were 23-fold higher than that of full-dose recipients [53].

Some studies showed that neutralizing antibody titers in COVID-19 were predictive of immune protection [54,55]. Neutralization titers continuously declined and appeared to be short-lived [56,57], and immune escape was observed in several SARS-CoV-2 variants of concern [58,59,60,61], leading to reduced VE to some extent [56]. The immune escape may cause a reduced VE among variants of concern, regardless of how long the last vaccination is given before the infection. A modelling meta-analysis study has previously demonstrated the correlation between neutralization titers with VE [56].

The magnitude of VE reduction depends on the initial effectiveness. The VE against symptomatic infection could, by day 250, drop to 77% or 33% if initial effectiveness was 95% or 70%, respectively [62]. Thus, the effectiveness of a vaccine may vary across types of vaccine, doses, and variants. The study by Khoury et al. [43] estimated that neutralizing antibody levels needed to protect against severe infection were six-fold lower than symptomatic infection, which could explain why the VE against severe infection in our study remained high, despite the low VE against symptomatic infection [62]. Since short-lived or substantial decay of neutralizing antibody titers means an increased vulnerability towards symptomatic infection, a persistent cellular immune memory enables a faster and stronger secondary immune response [63,64]. An appropriate secondary immune response, especially T-cell response, is protective against severe infection [65,66,67].

Our analyses showed a moderate VE reduction against the Omicron variant. Nonetheless, the Omicron variant did not display an increased severity despite the increased transmissibility [68,69,70]. Our results showed that VE estimates against severe infection still exhibit a high effectiveness for both the full and booster doses. However, we should be aware that new variants of concern may emerge anytime, and always need to be anticipated. Maintaining the COVID-19 pandemic to a low endemic level is seemingly a reasonable target before the eradication of COVID-19 can be achieved.

We acknowledge that this study has several limitations. First, most results had high heterogeneity. In the meta-analysis of VED outcome, we attempted to perform subgroup analyses based on the study design and types of the vaccine, but the heterogeneity remained high. Since there was a considerable discrepancy in the follow-up time between the booster and full doses, we suspected that the high heterogeneity was due to covariate time, as we have demonstrated in the other outcomes. Secondly, all included studies were observational studies. In observational studies, some confounding factors are difficult to measure, and therefore cannot be controlled; for example, significant differences in the follow-up time would result in different exposure received between the two groups. Moreover, the fact that VE declines over time should be considered because cumulative comparison would lead to a bias. As a result, we attempted to limit the time interval in one model to only include data within three months to minimize this bias. Third, some included studies were obtained from preprint servers, which had not been preceded by a peer-review process and the presented data may differ from the final, published, peer-reviewed version.

## 5. Conclusions

A lower initial VE supports the evidence that the SARS-CoV-2 Omicron variant has an increased immune escape ability, and the decline of VE over time suggests that the immunity to the SARS-CoV-2 Omicron variant infection is waned over time. The VE of the booster dose was generally higher than that of the full dose. A booster vaccination dose is recommended to confer the utmost protection against the SARS-CoV-2 Omicron variant infection. Moreover, the emergence of other variants of concern should always be anticipated. Nevertheless, these meta-analyses and meta-regression were constructed upon observational studies with different follow-up times, in which more extensive confounder adjustments could be difficult to perform. Therefore, future RCTs might be able to address several limitations of this study.

## Figures and Tables

**Table 1:** Characteristics of the included studies.

Reference	Study Design	County of Origin	Sample Sizes	Age /Years	Omicron Strain Confirmation Method	Follow-up Duration & (Median (IQR)) or Range) ^+^/Days		Type of Vaccines	Endpoints	Vaccine Effectiveness ^§^ /100%
						Dose 2	Dose 3			
Buchan et al. [23]	Case- control ^^^	Canada	134,435	≥18	Viral whole genome or S-gene sequencing	NR	NR	BNT162b2, mRNA-1273	Symptomatic infection and severe infection	1-OR
Gray et al. [24]	Case- control ^^^	South Africa	52,468	≥18	Omicron period	N/A	0–13 days group 8 (5–11) days 14–27 days group 20 (17–24) days 1–2 months group 32 (29–34) days	Ad26. COV2	Positive COVID-19 and severe infection	1-OR
Accorsi et al. [25]	Case- control ^^^	United States	70,155	≥18	Viral ORFlab, S, and N gene sequencing	8.0 (1.0) months	1.0 (1.0) month	BNT162b2, mRNA-1273	Symptomatic infection	1-OR
Andrews et al. [26]	Case-control ^^^	United Kingdom	2,663,549	≥18	Viral whole genome and S-gene sequencing	NR	39 (range, 14–118)	ChAdOx1, BNT162b2, mRNA-1273	Symptomatic infection	1-OR
Chemaitelly et al. [27]	Case- control ^^^	Qatar	133,327	No ≥ restriction	Viral whole genome sequencing	NR	NR	BNT162b2, mRNA-1273	Symptomatic infection	1-OR
Collie et al. [28]	Case- control ^^^	South Africa	211,610	≥18	Viral S-gene sequencing	NR	NR	BNT162b2	Symptomatic infection and severe infection	1-OR
Ferdinand et al. [29]	Case- control ^^^	United States	93,408	≥18	Omicron- predominance period	214 (164–259)	49 (30–73)	any mRNA vaccines ***	Symptomatic infection	1-OR
Klein et al. [30]	Case- control ^^^	United States	39,217	≥18	Omicron- predominance period	5 to 11 y.: 14–67 12–15 y.: NR 16–17 y.: NR	NR	BNT162b2	Symptomatic infection	1-OR
Lauring et al. [31]	Case- control ^^^	United States	11,690	≥18	Viral whole genome sequencing	NR	69.5 (41.5–97)	any mRNA vaccines ***	Severe infection	1-OR
Natarajan et al. [32]	Case- control ^^^	United States	80,287	≥18	Omicron- predominance period	Ad26. COV2.S: 52 (33–71) any mRNA: 48 (32–71)	Median (IQR) 59 (38–79)	any mRNA vaccines ***	Symptomatic infection and Hospitalization	1-OR
Tartof et al. [33]	Case- control ^^^	United States	14,137	≥18	Viral whole genome and S-gene sequencing	NR	NR	BNT162b2	Symptomatic infection and severe infection	1-OR
Tenforde et al. [34]	Case- control ^^^	United States	7544	≥18	Omicron- predominance period	256	60	any mRNA vaccines ***	Severe infection ^#^	1-OR
Thompson et al. [35]	Case- control ^^^	United States	222,772	≥18	Omicron- predominant period	<180 days group: 137 ≥180 days group: 223	Median interval: 41–44	any mRNA vaccines ***	Positive COVID-19 and severe infection	1-OR
Tseng et al. [36]	Case- control ^^^	United States	136,345	≥18	Viral whole genome and S-gene sequencing	14–365 days	NR	mRNA-1273	Positive COVID-19 and severe infection	1-OR
Young-Xu et al. [37]	Case- control ^^^	United States	69,215	≥18	Omicron- predominant period	NR	NR	any mRNA vaccines ***	Positive COVID-19	1-OR
Zambrano et al. [38]	Case- control ^^^	United States	283	12 to 18	Viral genome sequencing	MIS-C: 63 (48–89)	N/A	BNT162b2	Severe infection ^&^	1-OR
Abu-Raddad et al. [39]	Retrospective Cohort	Qatar	2,239,193	No restriction	Viral genome sequencing	21 (11–38)	22 (12–38)	BNT162b2, mRNA-1273	Symptomatic infection and severe infection	1-HR
Hansen et al. [40]	Retrospective Cohort	Denmark	5767	12 to 60	Sequencing of viral whole genome or a novel variant specific targeting the 452L mutation	1–150	1–30	BNT162b2, mRNA-1273	Positive COVID-19	1-HR
Monge et al. [41]	Retrospective Cohort	Spain	6,222,318	≥40	Omicron- predominant period	0–34	0–34	ChAdOx1-S, Ad26. COV2.S, mRNA-1273, BNT162b2	Positive COVID-19	1-RR
Fowlkes et al. [42]	Prospective cohort	United States	1364	5 to 18	Viral genome sequencing	5 to 11 y.: 14–82 12 to 15 y.: NR	N/A	BNT162b2	Any infection	1-HR

Data obtained from the emergency department and urgent care. ^^^ Test-negative case-control design. ^#^ Data from IMV or hospital-related death. ^&^ Multisystem Inflammatory Syndrome in children (MIS-C). ^+^ The number shows a median (IQR) or range between dose receipt date and endpoint date. The purpose is to depict how data distribution between booster and full dose differ. If the study did not report the limit of follow-up interval, then it would be written as not reported. A detailed outcome summary with 95%CI is compiled in Tables S5 and S6 for full and booster doses, respectively. Test-negative case-control study design. ^§^ 1–OR is 1–(odds among vaccinated group)/(odds among unvaccinated group); 1–RR is 1–(risk among vaccinated group)/(risk among unvaccinated group); 1–HR is 1–(hazard among vaccinated group)/(hazard among unvaccinated group)). *** any mRNA vaccine is designated for studies that describe the vaccines as mRNA vaccines but do not specify further the name of vaccines. Abbreviations: MIS-C, Multisystem Inflammatory Syndrome in children; N/A, not available; NR, not reported; y., year.

**Table 2:** VE estimates and VE reduction for booster dose and full dose of each vaccine.

Vaccine	Full Vaccination Dose			Booster Vaccination Dose		
	*n*	VE Estimate (%) (95%CI)	VE Reduction ^§^ (% per Month) (95%CI)	*n*	VE Estimate (%) (95%CI)	VE Reduction ^§^ (% per Month) (95%CI)
Any infection						
Overall Results	89	49.45 (38.01 to 60.88)	−2.45 (−4.26 to −0.63)	52	56.78 (47.11 to 66.45)	1.79 (−1.25 to 4.82)
Any mRNA	23	54.96 (30.63 to 79.29)	−0.93 (−4.64 to 2.78)	15	76.81 (59.99 to 93.63)	0.05 (−3.79 to 3.89)
Ad26.COV2.S	2	26.38 (−15.77 to 68.54)	N/A	8	48.66 (0.31 to 97)	0.58 (−19.47 to 20.62)
BNT162b2	39	52.15 (33.84 to 70.47)	−3.36 (−6.19 to −0.53)	17	53.04 (34.27 to 71.81)	1.82 (−4.61 to 8.24)
mRNA-1273	19	47.8 (25.48 to 70.12)	−2.98 (−6.44 to 0.47)	9	57.12 (30.57 to 83.68)	−0.88 (−15.27 to 13.5)
ChAdOx1 nCov-19	6	56.03 (47.81 to 64.25)	−8.53 (−9.98 to −7.09)	3	61.12 (25.33 to 96.91)	−5.87 (−27.76 to 16.02)
Symptomatic infection						
Overall Results	53	56.36 (47.21 to 65.51)	−5.5 (−7.01 to −3.99)	32	53.58 (44.18 to 62.98)	1.14 (−1.99 to 4.26)
Any mRNA	11	51.69 (22.71 to 80.68)	−3.3 (−7.86 to 1.25)	7	63.93 (30.38 to 97.47)	0.89 (−5.97 to 7.74)
Ad26.COV2.S	1	24 (18.5 to 29.5)	N/A	1	54 (44 to 64)	N/A
BNT162b2	26	58.24 (45.84 to 70.64)	−5.8 (−7.69 to −3.91)	14	55.65 (39.04 to 72.25)	−1.68 (−8 to 4.65)
mRNA-1273	9	79.61 (68.83 to 90.39)	−10.76 (−12.91 to −8.6)	7	52.88 (8.61 to 97.15)	0.91 (−37.41 to 39.23)
ChAdOx1 nCov-19	6	56.03 (47.81 to 64.25)	−8.53 (−9.98 to −7.09)	3	61.12 (25.33 to 96.91)	−5.87 (−27.76 to 16.02)
Severe infection						
Overall Results	21	64.81 (49.9 to 79.73)	0.59 (−1.49 to 2.67)	13	92.53 (85.54 to 99.52)	−1.27 (−3.07 to 0.53)
Any mRNA	12	64.3 (46.19 to 82.42)	0.72 (−2 to 3.43)	7	94.54 (90.94 to 98.14)	−1.24 (−2.11 to −0.38)
Ad26.COV2.S	1	31 (21.5 to 40.5)	N/A	4	81.74 (38.01 to 125.46)	−3.02 (−20.41 to 14.37)
BNT162b2	6	80.17 (55.61 to 104.73)	−0.58 (−3.72 to 2.56)	2	89.05 (86.39 to 91.71)	N/A
mRNA-1273	2	66.86 (−1.23 to 134.94)	N/A			

^§^ VE reduction per month is approximated by multiplying the VE reduction per day—the slope of VE vs. time—by 30. Abbreviations: *n*, number of analyses; N/A, not available; VE, vaccine effectiveness.

## Data Availability

Detailed methods, results, and additional data are available in the manuscript and the associated supplementary materials.

## Supplementary Materials

The following supporting information can be downloaded at: https://www.mdpi.com/article/10.3390/vaccines10122180/s1, Table S1: Results of systematic searches; Table S2: The assessment of quality of study for cohort studies using NOS; Table S3: The assessment of quality of study for case-control studies using NOS; Table S4: Summary of outcomes for full dose; Table S5: Summary of outcomes for booster dose; Table S6: Summary of outcomes for booster vs full dose of the within 3 months or more model; Table S7: Summary of outcomes for booster vs full dose of the within 3 months model; Figure S1: Sensitivity Analysis for Any Infection Endpoints of 0 to 3 Months Model; Figure S2: Sensitivity Analysis for Symptomatic Infection Endpoints of 0 to 3 Months Model; Figure S3: Sensitivity Analysis for Severe Infection Endpoints of 0 to 3 Months Model; Figure S4: Sensitivity Analysis for Any Infection Endpoints of 0 to 3 Months or More Model; Figure S5: Sensitivity Analysis for Symptomatic Infection Endpoints of 0 to 3 Months or More Model; Figure S6: Sensitivity Analysis for Severe Infection Endpoints of 0 to 3 Months or More Model.
