# Supplementary material for: Effectiveness of COVID-19 Vaccines against SARS-CoV-2 Omicron Variant (B.1.1.529): A Systematic Review with Meta-Analysis and Meta-Regression
Source: Vaccines (Basel). 2022 Dec 19;10(12):2180. doi: 10.3390/vaccines10122180 (PMC9783108; doi:10.3390/vaccines10122180)

SUPPLEMENTARY MATERIALS

Contents

S.1. Literature Search 2
The Keywords 2
The Search Results 2
Table S1. Results of systematic searches. 2
S.2. Assessment of Quality of Study 3
Table S2. The assessment of quality of study for cohort studies using Newcastle-Ottawa Scale (NOS) 3
Table S3. The assessment of quality of study for case-control studies using Newcastle-Ottawa Scale (NOS) 5
S.3. Summary of outcomes for full dose 7
Table S4. Summary of outcomes for full dose 7
S.4. Summary of outcomes for booster dose 11
Table S5. Summary of outcomes for booster dose 11
S.5. Summary of outcomes for booster vs. full dose of the within 3 months or more model 14
Table S6. Summary of outcomes for booster vs. full dose of the within 3 months or more model 14
S.6. Summary of outcomes for booster vs. full dose of the within 3 months model 15
Table S7. Summary of outcomes for booster vs. full dose of the within 3 months model . 15
Figure S1. Sensitivity Analysis for Any Infection Endpoints of 0 to 3 Months Model 17
Figure S2. Sensitivity Analysis for Symptomatic Infection Endpoints of 0 to 3 Months Model 18
Figure S3. Sensitivity Analysis for Severe Infection Endpoints of 0 to 3 Months Model 19
Figure S4. Sensitivity Analysis for Any Infection Endpoints of 0 to 3 Months or More Model 20
Figure S5. Sensitivity Analysis for Symptomatic Infection Endpoints of 0 to 3 Months or More Model 21
Figure S6. Sensitivity Analysis for Severe Infection Endpoints of 0 to 3 Months or More Model 22

S.1. Literature Search

The Keywords

#1 (SARS-CoV-2) OR (COVID-19)

#2 (Omicron) OR (B.1.1.529)

#3 (Vaccine) OR (Vaccination)

#4 (Vaccine efficacy) OR (Vaccine effectiveness)

The Search Results

Table S1. Results of systematic searches.

Database	Keywords	Search Result	Search-time
PubMed	#1 AND #2 AND #3 AND #4	121	April 6th, 2022
ScienceDirect	#1 AND #2 AND #3 AND #4	475	April 6th, 2022
CENTRAL	#1 AND #2 AND #3 AND #4	2	April 6th, 2022
Web of Science	#1 AND #2 AND #3 AND #4	61	April 6th, 2022
Scopus	#1 AND #2 AND #3 AND #4	619	April 6th, 2022
medRxiv	#1 AND #2 AND #3 AND #4	455	April 6th, 2022
bioRxiv	#1 AND #2 AND #3 AND #4	312	April 6th, 2022
Bibliographic search	-	19	April 6th, 2022

S.2. Assessment of Quality of Study

Table S2. The assessment of quality of study for cohort studies using Newcastle-Ottawa Scale (NOS)

**NOS of Cohort Study**				
**Components**	Hansen et al. (2021)	Abu-Raddad et al. (2022)	Monge et al., (2022)	Fowlkes et al. (2022)
**Selection**				
Representativeness of the exposed cohort	*	*	*	*
Selection of the non-exposed cohort	*	*	*	*
Ascertainment of exposure	*	*	*	*
Demonstration that outcome of interest was not present at start of the study	*	*	-	-
**Comparability**				
Comparability of cohorts on the basis of design or analysis	*	**	*	*
**Exposure**				
Assessment of outcome	*	*	*	*

Enough follow-up time length for outcome to occur	*	*	*	*
Adequacy of follow-up of cohorts	*	*	*	*
**Study Quality**				
**Total Score**	8	9	7	7
**Interpretation**	Good	Good	Good	Good

Table S3. The assessment of quality of study for case-control studies using Newcastle-Ottawa Scale (NOS)

NOS of Case-Control Study										
	**Selection**				**Comparability**	**Exposure**			**Study Quality**	
**Components**	Is the case definition adequate?	Representative-ness of the cases	Selection of controls	Definition of controls	Comparability of cases and controls on the basis of the design or analysis	Ascertainment of exposure	Same method of ascertainment for cases and controls	Non-response rate	**Total Score**	**Interpretation**
Buchan et al. (2021)	*	*	*	*	*	-	*	*	7	Good
Gray et al. (2021)	*	*	*	*	*	*	*	*	8	Good
Accorsi et al. (2022)	*	*	*	*	*	-	*	*	7	Good
Andrews et al. (2022)	*	*	*	*	*	*	*	*	8	Good
Chemaitelly et al. (2022)	*	*	*	*	*	*	*	*	8	Good
Collie et al. (2022)	*	*	*	*	*	*	*	*	8	Good
Ferdinand et al. (2022)	*	*	*	*	*	*	*	*	8	Good
Klein et al. (2022)	*	*	*	*	*	*	*	*	8	Good
Lauring et al. (2022)	*	*	*	*	**	*	*	*	9	Good
Natarajan et al. (2022)	*	*	*	*	**	*	*	*	9	Good
Tartof et al. (2022)	*	*	*	*	*	*	*	*	8	Good
Tenforde et al. (2022)	*	*	*	*	*	-	*	*	7	Good

Thompson et al. (2022)	*	*	*	*	*	*	*	*	8	Good
Tseng et al. (2022)	*	*	*	*	**	*	*	*	9	Good
Young-Xu et al. (2022)	*	*	*	*	*	*	*	*	8	Good
Zambrano et al. (2022)	*	*	*	*	*	*	*	*	8	Good

S.3. Summary of outcomes for full dose

Table S4. Summary of outcomes for full dose

Study	Endpoints	Vaccine	Study Design	Days Latitude	Follow-up Interval	VE (95%CI)
Ferdinand et al. (2022)	Severe infection	Any mRNA	Case-control	60	0 to 2 months	71% (51% to 83%)
Ferdinand et al. (2022)	Severe infection	Any mRNA	Case-control	90	2 to 3 months	65% (53% to 74%)
Ferdinand et al. (2022)	Severe infection	Any mRNA	Case-control	120	3 to 4 months	58% (38% to 71%)
Ferdinand et al. (2022)	Severe infection	Any mRNA	Case-control	150	5+ months	54% (48% to 59%)
Ferdinand et al. (2022)	Symptomatic Infection	Any mRNA	Case-control	60	0 to 2 months	69% (62% to 75%)
Ferdinand et al. (2022)	Symptomatic Infection	Any mRNA	Case-control	90	2 to 3 months	50% (45% to 55%)
Ferdinand et al. (2022)	Symptomatic Infection	Any mRNA	Case-control	120	3 to 4 months	48% (41% to 54%)
Ferdinand et al. (2022)	Symptomatic Infection	Any mRNA	Case-control	150	5+ months	37% (34% to 40%)
Klein et al. (2022)	Symptomatic infection	BNT162b2	Case-control	149	14 to 149 days	34% (8% to 53%)
Klein et al. (2022)	Symptomatic infection	BNT162b2	Case-control	299	150+ days	-3% (-30% to 18%)
Klein et al. (2022)	Symptomatic infection	BNT162b2	Case-control	149	14 to 149 days	45% (30% to 57%)
Klein et al. (2022)	Symptomatic infection	BNT162b2	Case-control	299	150+ days	-2% (-25% to 17%)
Klein et al. (2022)	Symptomatic infection	BNT162b2	Case-control	67	14 to 67 days	51% (30% to 65%)
Natarajan et al. (2022)	Severe infection	AD26.COV2S	Case-control	120	14+ days	31% (21% to 40%)
Natarajan et al. (2022)	Symptomatic Infection	AD26.COV2S	Case-control	120	14+ days	24% (18% to 29%)
Tenforde et al. (2022)	Severe infection	Any mRNA	Case-control	256	256 days (median)	79% (66% to 87%)
Zambrano et al. (2022)	Severe Infection	BNT162b2	Case-control	63	63 days (median)	90% (75% to 96%)
Andrews et al. (2022)	Symptomatic Infection	ChAdOx1 nCov-19	Case-control	28	2 to 4 weeks	48.9% (39.2% to 57.1%)
Andrews et al. (2022)	Symptomatic Infection	ChAdOx1 nCov-19	Case-control	70	5 to 9 weeks	33.7% (25% to 41.5%)
Andrews et al. (2022)	Symptomatic Infection	ChAdOx1 nCov-19	Case-control	105	10 to 14 weeks	28.6% (20.9% to 35.6%)
Andrews et al. (2022)	Symptomatic Infection	ChAdOx1 nCov-19	Case-control	140	15 to 19 weeks	17.8% (13.4% to 21.9%)
Andrews et al. (2022)	Symptomatic Infection	ChAdOx1 nCov-19	Case-control	175	20 to 24 weeks	4% (1.9% to 6.1%)
Andrews et al. (2022)	Symptomatic Infection	ChAdOx1 nCov-19	Case-control	210	25+ weeks	-2.7% (-4.2% to -1.2%)
Andrews et al. (2022)	Symptomatic Infection	BNT162b2	Case-control	28	2 to 4 weeks	65.5% (63.9% to 67%)
Andrews et al. (2022)	Symptomatic Infection	BNT162b2	Case-control	70	5 to 9 weeks	48.7% (47.1% to 50.2%)
Andrews et al. (2022)	Symptomatic Infection	BNT162b2	Case-control	105	10 to 14 weeks	30.1% (28.7% to 31.5%)

Andrews et al. (2022)	Symptomatic Infection	BNT162b2	Case-control	140	15 to 19 weeks	15.4% (14.2% to 16.6%)
Andrews et al. (2022)	Symptomatic Infection	BNT162b2	Case-control	175	20 to 24 weeks	11.5% (10.1% to 12.9%)
Andrews et al. (2022)	Symptomatic Infection	BNT162b2	Case-control	210	25+ weeks	8.8% (7% to 10.5%)
Andrews et al. (2022)	Symptomatic Infection	mRNA-1273	Case-control	28	2 to 4 weeks	75.1% (70.8% to 78.7%)
Andrews et al. (2022)	Symptomatic Infection	mRNA-1273	Case-control	70	5 to 9 weeks	52.8% (48.2% to 57.1%)
Andrews et al. (2022)	Symptomatic Infection	mRNA-1273	Case-control	105	10 to 14 weeks	35.6% (32.7% to 38.4%)
Andrews et al. (2022)	Symptomatic Infection	mRNA-1273	Case-control	140	15 to 19 weeks	25.3% (23.2% to 27.4%)
Andrews et al. (2022)	Symptomatic Infection	mRNA-1273	Case-control	175	20 to 24 weeks	15% (11.6% to 18.2%)
Andrews et al. (2022)	Symptomatic Infection	mRNA-1273	Case-control	210	25+ weeks	14.9% (3.9% to 24.7%)
Buchan et al. (2021)	Symptomatic Infection	Any mRNA	Case-control	59	7 to 59 days	36% (24% to 45%)
Buchan et al. (2021)	Symptomatic Infection	Any mRNA	Case-control	119	60 to 119 days	12% (3% to 21%)
Buchan et al. (2021)	Symptomatic Infection	Any mRNA	Case-control	179	120 to 179 days	15% (8% to 22%)
Buchan et al. (2021)	Symptomatic Infection	Any mRNA	Case-control	239	180 to 239 days	1% (-8% to 10%)
Buchan et al. (2021)	Symptomatic Infection	Any mRNA	Case-control	299	240+ days	2% (-17% to 17%)
Buchan et al. (2021)	Severe infection	Any mRNA	Case-control	59	7 to 59 days	55% (-106% to 90%)
Buchan et al. (2021)	Severe infection	Any mRNA	Case-control	119	60 to 119 days	37% (-71% to 77%)
Buchan et al. (2021)	Severe infection	Any mRNA	Case-control	179	120 to 179 days	75% (51% to 87%)
Buchan et al. (2021)	Severe infection	Any mRNA	Case-control	239	180 to 239 days	82% (62% to 91%)
Buchan et al. (2021)	Severe infection	Any mRNA	Case-control	299	240+ days	86% (-12% to 98%)
Chemaitelly et al. (2022)	Symptomatic Infection	BNT162b2	Case-control	30	0 to 1 month	61.9% (49.9% to 71.1%)
Chemaitelly et al. (2022)	Symptomatic Infection	BNT162b2	Case-control	60	1 to 2 months	45.9% (33.8% to 55.8%)
Chemaitelly et al. (2022)	Symptomatic Infection	BNT162b2	Case-control	90	2 to 3 months	36.3% (25.1% to 45.8%)
Chemaitelly et al. (2022)	Symptomatic Infection	BNT162b2	Case-control	120	3 to 4 months	28.5% (18% to 37.8%)
Chemaitelly et al. (2022)	Symptomatic Infection	BNT162b2	Case-control	150	4 to 5 months	10.6% (-2.3% to 21.9%)
Chemaitelly et al. (2022)	Symptomatic Infection	BNT162b2	Case-control	180	5 to 6 months	14.3% (6.2% to 21.8%)
Chemaitelly et al. (2022)	Symptomatic Infection	BNT162b2	Case-control	210	6 to 7 months	9.6% (2.4% to 16.3%)
Chemaitelly et al. (2022)	Symptomatic Infection	BNT162b2	Case-control	240	7 to 8 months	-7.5% (-15.3% to -0.2%)
Chemaitelly et al. (2022)	Symptomatic Infection	BNT162b2	Case-control	270	8 to 9 months	1.5% (-6.2% to 8.7%)
Chemaitelly et al. (2022)	Symptomatic Infection	BNT162b2	Case-control	300	9 to 10 months	-17.7% (-25.6% to -10.3%)
Chemaitelly et al. (2022)	Symptomatic Infection	BNT162b2	Case-control	330	10 to 11 months	-0.3% (-10.2% to 8.6%)

Chemaitelly et al. (2022)	Symptomatic Infection	BNT162b2	Case-control	360	11+ months	16.5% (3.1% to 28.1%)
Chemaitelly et al. (2022)	Symptomatic Infection	mRNA-1273	Case-control	120	1 to 3 months	44.8% (16% to 63.8%)
Chemaitelly et al. (2022)	Symptomatic Infection	mRNA-1273	Case-control	180	4 to 6 months	20.8% (13.7% to 27.4%)
Chemaitelly et al. (2022)	Symptomatic Infection	mRNA-1273	Case-control	240	7+ months	-9.3% (-16.3% to -2.8%)
Chemaitelly et al. (2022)	Severe infection	mRNA-1273	Case-control	180	1 to 6 months	76.9% (19.2% to 93.4%)
Chemaitelly et al. (2022)	Severe infection	mRNA-1273	Case-control	360	7+ months	64% (39.1% to 78.7%)
Chemaitelly et al. (2022)	Severe infection	BNT162b2	Case-control	180	1 to 6 months	73.7% (46.8% to 87%)
Chemaitelly et al. (2022)	Severe infection	BNT162b2	Case-control	360	7+ months	80.7% (71.3% to 87%)
Tartof et al. (2022)	Severe Infection	BNT162b2	Case-control	90	0 to 3 months	70% (41% to 84%)
Tartof et al. (2022)	Severe Infection	BNT162b2	Case-control	180	3 to 6 months	67% (44% to 80%)
Tartof et al. (2022)	Severe Infection	BNT162b2	Case-control	270	6+ months	68% (56% to 76%)
Tartof et al. (2022)	Symptomatic Infection	BNT162b2	Case-control	90	0 to 3 months	60% (43% to 72%)
Tartof et al. (2022)	Symptomatic Infection	BNT162b2	Case-control	180	3 to 6 months	38% (21% to 51%)
Tartof et al. (2022)	Symptomatic Infection	BNT162b2	Case-control	270	6+ months	41% (32% to 50%)
Thompson et al. (2022)	Symptomatic Infection	Any mRNA	Case-control	180	14 to 179 days	52% (46% to 58%)
Thompson et al. (2022)	Symptomatic Infection	Any mRNA	Case-control	360	180+ days	38% (32% to 43%)
Thompson et al. (2022)	Severe Infection	Any mRNA	Case-control	179	14 to 179 days	81% (65% to 90%)
Thompson et al. (2022)	Severe Infection	Any mRNA	Case-control	359	180+ days	57% (39% to 70%)
Tseng et al. (2022)	Positive Covid-19	mRNA-1273	Case-control	90	14 to 90 days	44% (35.1% to 51.6%)
Tseng et al. (2022)	Positive Covid-19	mRNA-1273	Case-control	180	91 to 180 days	23.5% (16.4% to 30%)
Tseng et al. (2022)	Positive Covid-19	mRNA-1273	Case-control	270	181 to 270 days	13.8% (10.2% to 17.3%)
Tseng et al. (2022)	Positive Covid-19	mRNA-1273	Case-control	365	271 to 365 days	5.9% (0.4% to 11%)
Fowlkes et al. (2022)	Positive Covid-19	BNT162b2	Cohort	82	14 to 82 days	31% (9% to 48%)
Fowlkes et al. (2022)	Positive Covid-19	BNT162b2	Cohort	149	14 to 149 days	59% (22% to 79%)
Fowlkes et al. (2022)	Positive Covid-19	BNT162b2	Cohort	299	150+ days	62% (-28% to 89%)
Hansen et al. (2021)	Positive Covid-19	BNT162b2	Cohort	30	1 to 30 days	55.2% (23.5% to 73.7%)
Hansen et al. (2021)	Positive Covid-19	BNT162b2	Cohort	60	31 to 60 days	16.1% (-20.8% to 41.7%)
Hansen et al. (2021)	Positive Covid-19	BNT162b2	Cohort	90	61 to 90 days	9.8% (-10% to 26.1%)
Hansen et al. (2021)	Positive Covid-19	BNT162b2	Cohort	150	91 to 150 days	-76.2% (-95.3% to 59.5%)
Hansen et al. (2021)	Positive Covid-19	mRNA-1273	Cohort	30	1 to 30 days	36.7% (-69.9% to 76.4%)

Hansen et al. (2021)	Positive Covid-19	mRNA-1273	Cohort	60	31 to 60 days	30% (-41.3% to 65.4%)
Hansen et al. (2021)	Positive Covid-19	mRNA-1273	Cohort	90	61 to 90 days	4.2% (-30.8% to 29.8%)
Hansen et al. (2021)	Positive Covid-19	mRNA-1273	Cohort	150	91 to 150 days	39.3% (-61.6% to -20%)

Data whose Days Latitude are day 14 or below and NR are excluded from the meta-regression analysis

S.4. Summary of outcomes for booster dose

Table S5. Summary of outcomes for booster dose

Study	Endpoints	Vaccine	Study Design	Days Latitude	Follow-up Interval	VE (95%CI)
Buchan et al., 2021	Severe infection	Any mRNA	Case-control	6	0 to 6 days	91% (71% to 97%)
Buchan et al., 2021	Symptomatic Infection	Any mRNA	Case-control	6	0 to 6 days	36% (29% to 43%)
Andrews et al., 2022	Symptomatic Infection	BNT162b2	Case-control	7	1 week	66.9% (66.1% to 67.6%)
Andrews et al., 2022	Symptomatic Infection	ChAdOx1 nCov-19	Case-control	7	1 week	57.7% (37.6% to 71.3%)
Andrews et al., 2022	Symptomatic Infection	mRNA-1273	Case-control	7	1 week	68.1% (65.6% to 70.5%)
Chemaitelly et al., 2022	Symptomatic Infection	BNT162b2	Case-control	7	1 week	15.8% (0.9% to 28.4%)
Chemaitelly et al., 2022	Symptomatic Infection	mRNA-1273	Case-control	7	1 week	3.6% (-31% to 29.1%)
Gray et al., 2021	Severe Infection	AD26.COV2S	Case-control	13	0 to 13 days	63% (31% to 81%)
Buchan et al., 2021	Severe infection	Any mRNA	Case-control	14	7+ days	95% (87% to 98%)
Buchan et al., 2021	Symptomatic Infection	Any mRNA	Case-control	14	7+ days	61% (56% to 65%)
Gray et al., 2021	Positive Covid-19	AD26.COV2S	Case-control	14	0 to 13 days	31.9% (31.5% to 32.2%)
Chemaitelly et al., 2022	Symptomatic Infection	BNT162b2	Case-control	21	2 to 3 weeks	53.6% (47.4% to 59.1%)
Chemaitelly et al., 2022	Symptomatic Infection	mRNA-1273	Case-control	21	2 to 3 weeks	53.1% (40.7% to 62.8%)
Gray et al., 2021	Severe Infection	AD26.COV2S	Case-control	27	14 to 27 days	84% (67% to 92%)
Gray et al., 2021	Positive Covid-19	AD26.COV2S	Case-control	28	14 to 27 days	14.4% (14% to 14.6%)
Accorsi et al., 2022	Symptomatic Infection	BNT162b2	Case-control	30	14+ days	65% (62% to 68%)
Accorsi et al., 2022	Symptomatic Infection	mRNA-1273	Case-control	30	14+ days	72% (69% to 74%)
Monge et al., 2022	Positive Covid-19	Any mRNA	Case-control	34	7 to 34 days	50.4% (46% to 54.8%)
Andrews et al., 2022	Symptomatic Infection	BNT162b2	Case-control	35	2 to 4 weeks	67.2% (66.5% to 67.8%)
Andrews et al., 2022	Symptomatic Infection	ChAdOx1 nCov-19	Case-control	35	2 to 4 weeks	55.6% (44.4% to 64.6%)
Andrews et al., 2022	Symptomatic Infection	mRNA-1273	Case-control	35	2 to 4 weeks	66.3% (63.7% to 68.8%)
Chemaitelly et al., 2022	Symptomatic Infection	BNT162b2	Case-control	42	4 to 5 weeks	56.6% (50.8% to 61.7%)

Chemaitelly et al., 2022	Symptomatic Infection	mRNA-1273	Case-control	42	4 to 5 weeks	54.6% (41.1% to 65%)
Gray et al., 2021	Severe Infection	AD26.COV2S	Case-control	56	28 to 56 days	85% (54% to 95%)
Ferdinand et al., 2022	Severe infection	Any mRNA	Case-control	60	0 to 2 months	91% (88% to 93%)
Ferdinand et al., 2022	Symptomatic Infection	Any mRNA	Case-control	60	0 to 2 months	87% (85% to 88%)
Tenforde et al., 2022	Severe infection	Any mRNA	Case-control	60	60 days (median)	94% (88% to 97%)
Tseng et al., 2022	Positive Covid-19	mRNA-1273	Case-control	60	14 to 60 days	71.6% (69.7% to 73.4%)
Chemaitelly et al., 2022	Symptomatic Infection	BNT162b2	Case-control	63	6 to 7 weeks	46.2% (39.7% to 52%)
Chemaitelly et al., 2022	Symptomatic Infection	mRNA-1273	Case-control	63	6+ weeks	38.6% (19.4% to 53.1%)
Klein et al., 2022	Symptomatic Infection (Age 5-11 years)	BNT162b2	Case-control	67	14 to 67 days	81% (59% to 91%)
Andrews et al., 2022	Symptomatic Infection	BNT162b2	Case-control	70	5 to 9 weeks	55% (54.2% to 55.8%)
Andrews et al., 2022	Symptomatic Infection	ChAdOx1 nCov-19	Case-control	70	5 to 9 weeks	46.7% (34.3% to 56.7%)
Chemaitelly et al., 2022	Symptomatic Infection	BNT162b2	Case-control	84	8 to 9 weeks	38% (28.1% to 46.5%)
Gray et al., 2021	Positive Covid-19	AD26.COV2S	Case-control	87	27 to 28 days	6.1% (5.1% to 7%)
Tartof et al., 2022	Severe infection	BNT162b2	Case-control	90	0 to 3 months	89% (83% to 92%)
Tartof et al., 2022	Symptomatic Infection	BNT162b2	Case-control	90	6+ months	78% (73% to 82%)
Andrews et al., 2022	Symptomatic Infection	BNT162b2	Case-control	105	10+ week	45.7% (44.7% to 46.7%)
Chemaitelly et al., 2022	Symptomatic Infection	BNT162b2	Case-control	105	10 to 11 weeks	43.7% (32.9% to 52.7%)
Natarajan et al., 2022	Severe infection	AD26.COV2S	Case-control	120	7 to 120 days	67% (52% to 77%)
Natarajan et al., 2022	Severe infection	Any mRNA	Case-control	120	7 to 120 days	89.5% (84.2% to 94.8%)
Natarajan et al., 2022	Symptomatic Infection	AD26.COV2S	Case-control	120	7 to 120 days	54% (43% to 63%)

Natarajan et al., 2022	Symptomatic Infection	Any mRNA	Case-control	120	7 to 120 days	82.7% (80.1% to 85.3%)
Tseng et al., 2022	Positive Covid-19	mRNA-1273	Case-control	120	60+ days	47.4% (40.5% to 53.5%)
Chemaitelly et al., 2022	Symptomatic Infection	BNT162b2	Case-control	126	12+ weeks	37.6% (28.8% to 45.4%)
Ferdinand et al., 2022	Severe infection	Any mRNA	Case-control	180	2 to 3 months	88% (85% to 90%)
Ferdinand et al., 2022	Symptomatic Infection	Any mRNA	Case-control	180	2 to 3 months	81% (79% to 82%)
Tartof et al., 2022	Severe infection	BNT162b2	Case-control	180	3 to 5 months	90% (57% to 98%)
Tartof et al., 2022	Symptomatic Infection	BNT162b2	Case-control	180	270+ days	48% (14% to 69%)
Ferdinand et al., 2022	Severe infection	Any mRNA	Case-control	240	4+ months	78% (67% to 85%)
Ferdinand et al., 2022	Symptomatic Infection	Any mRNA	Case-control	240	3 to 4 months	66% (59% to 71%)
Ferdinand et al., 2022	Symptomatic Infection	Any mRNA	Case-control	300	5+ months	31% (-50% to 68%)

Data whose Days Latitude are day 14 or below and NR are excluded from the meta-regression analysis

S.5. Summary of outcomes for booster vs full dose of the within 3 months or more model

Table S6. Summary of outcomes for booster vs. full dose of the within 3 months or more model

Authors	Outcomes	Design	Vaccine	VED (95% CI)
Andrews et al., 2022	Symptomatic Infection	Cohort	ChAdOx1 nCov-19	16.11% (2.41 to 29.82%)
Andrews et al., 2022	Symptomatic Infection	Cohort	BNT162b2	17.64% (16.24 to 19.05%)
Andrews et al., 2022	Symptomatic Infection	Cohort	mRNA-1273	16.52% (12.45 to 20.59%)
Buchan et al., 2021	Symptomatic Infection	Case-control	Any mRNA	27.25% (-5.06 to 59.56%)
Chemaitelly et al., 2022	Symptomatic Infection	Case-control	BNT162b2	0% (-6.08 to 6.08%)
Chemaitelly et al., 2022	Symptomatic Infection	Case-control	mRNA-1273	33% (-21.38 to 87.38%)
Tartof et al., 2022	Symptomatic Infection	Case-control	BNT162b2	-5.72% (-19.59 to 8.15%)
Abu-Raddad et al., 2022	Symptomatic Infection	Cohort	BNT162b2	-7.7% (-38.69 to 23.29%)
Abu-Raddad et al., 2022	Symptomatic Infection	Cohort	mRNA-1273	2% (-8.26 to 12.26%)
Natarajan et al., 2022	Symptomatic Infection	Case-control	Any mRNA	27.6% (19.15 to 36.06%)
Klein et al., 2022	symptomatic Infection	Case-control	BNT162b2	33% (23.82 to 42.18%)
Ferdinand et al., 2022	symptomatic Infection	Case-control	Any mRNA	19% (-2.97 to 40.97%)
Buchan et al., 2021	Severe infection	Case-control	Any mRNA	-3.1% (-6.29 to 0.09%)
Lauring et al., 2022	Severe infection	Case-control	Any mRNA	-3.5% (-6.45 to -0.55%)
Tartof et al., 2022	Severe infection	Case-control	BNT162b2	42% (32 to 52%)
Abu-Raddad et al., 2022	Severe infection	Cohort	BNT162b2	50.6% (48.35 to 52.85%)
Natarajan et al., 2022	Severe infection	Case-control	Any mRNA	23.5% (7.7 to 39.3%)
Tenforde et al., 2022	Severe infection	Case-control	Any mRNA	52.7% (46.4 to 59%)
Hansen et al., 2022	Positive Covid-19	Cohort	BNT162b2	29% (18.95 to 39.05%)
Tseng et al., 2022	Positive Covid-19	Case-control	mRNA-1273	17.5% (7.26 to 27.74%)
Monge et al., 2022	Positive Covid-19	Case-control	mRNA-1273	15% (3.58 to 26.42%)
Monge et al., 2022	Positive Covid-19	Case-control	BNT162b2	30% (12.93 to 47.07%)

S.6. Summary of outcomes for booster vs full dose of the within 3 months model

Table S7. Summary of outcomes for booster vs. full dose of the within 3 months model

Authors	Outcomes	Design	Vaccine	VED (95%CI)
Abu-Raddad et al., 2022	Symptomatic Infection	Cohort	BNT162b2	50.6% (50.18% to 51.02%)
Abu-Raddad et al., 2022	Symptomatic Infection	Cohort	mRNA-1273	52.7% (51.53% to 53.87%)
Abu-Raddad et al., 2022	Severe infection	Cohort	BNT162b2	23.5% (20.57% to 26.43%)
Accorsi et al., 2022	Symptomatic Infection	Case-control	BNT162b2	64.9% (64.34% to 65.46%)
Accorsi et al., 2022	Symptomatic Infection	Case-control	mRNA-1273	59.5% (58.81% to 60.19%)
Andrews et al., 2022	Symptomatic Infection	Cohort	ChAdOx1 nCov-19	27.29% (16.16% to 38.43%)
Andrews et al., 2022	Symptomatic Infection	Cohort	BNT162b2	28.15% (26.5% to 29.79%)
Andrews et al., 2022	Symptomatic Infection	Cohort	mRNA-1273	33.96% (28.34% to 39.59%)
Buchan et al., 2021	Symptomatic Infection	Case-control	Any mRNA	35.85% (24.81% to 46.89%)
Buchan et al., 2021	Severe infection	Case-control	Any mRNA	24.21% (-33.37% to 81.79%)
Chemaitelly et al., 2022	Symptomatic Infection	Case-control	mRNA-1273	6% (-14.52% to 26.51%)
Chemaitelly et al., 2022	Symptomatic Infection	Case-control	BNT162b2	9.01% (-23.82% to 41.83%)
Ferdinand et al., 2022	symptomatic Infection	Case-control	Any mRNA	22.33% (16.9% to 27.77%)
Hansen et al., 2022	Positive Covid-19	Cohort	BNT162b2	78.54% (53.26% to 103.81%)
Klein et al., 2022	symptomatic Infection	Case-control	BNT162b2	41.7% (21.43% to 61.98%)
Lauring et al., 2022	Severe infection	Case-control	Any mRNA	2% (-4.88% to 8.88%)
Natarajan et al., 2022	Symptomatic Infection	Case-control	Any mRNA	29% (26.41% to 31.59%)
Natarajan et al., 2022	Severe infection	Case-control	Any mRNA	17.5% (14.87% to 20.13%)
Tartof et al., 2022	Symptomatic Infection	Case-control	BNT162b2	33% (24.98% to 41.02%)
Tartof et al., 2022	Severe infection	Case-control	BNT162b2	19% (-0.18% to 38.18%)
Tenforde et al., 2022	Severe infection	Case-control	Any mRNA	15% (12.88% to 17.12%)
Thompson et al., 2022	Severe infection	Case-control	Any mRNA	9% (-1.9% to 19.9%)

Thompson et al., 2022	Positive Covid-19	Case-control	Any mRNA	30% (25.07% to 34.93%)
Tseng et al., 2022	severe infection	Case-control	mRNA-1273	14.7% (7.49% to 21.91%)
Tseng et al., 2022	Positive Covid-19	Case-control	mRNA-1273	27.6% (20.4% to 34.8%)
Yong-Xu et al., 2022	Positive Covid-19	Case-control	Any mRNA	21% (20.14% to 21.86%)

Figure S1. Sensitivity Analysis for Any Infection Endpoints of 0 to 3 Months Model

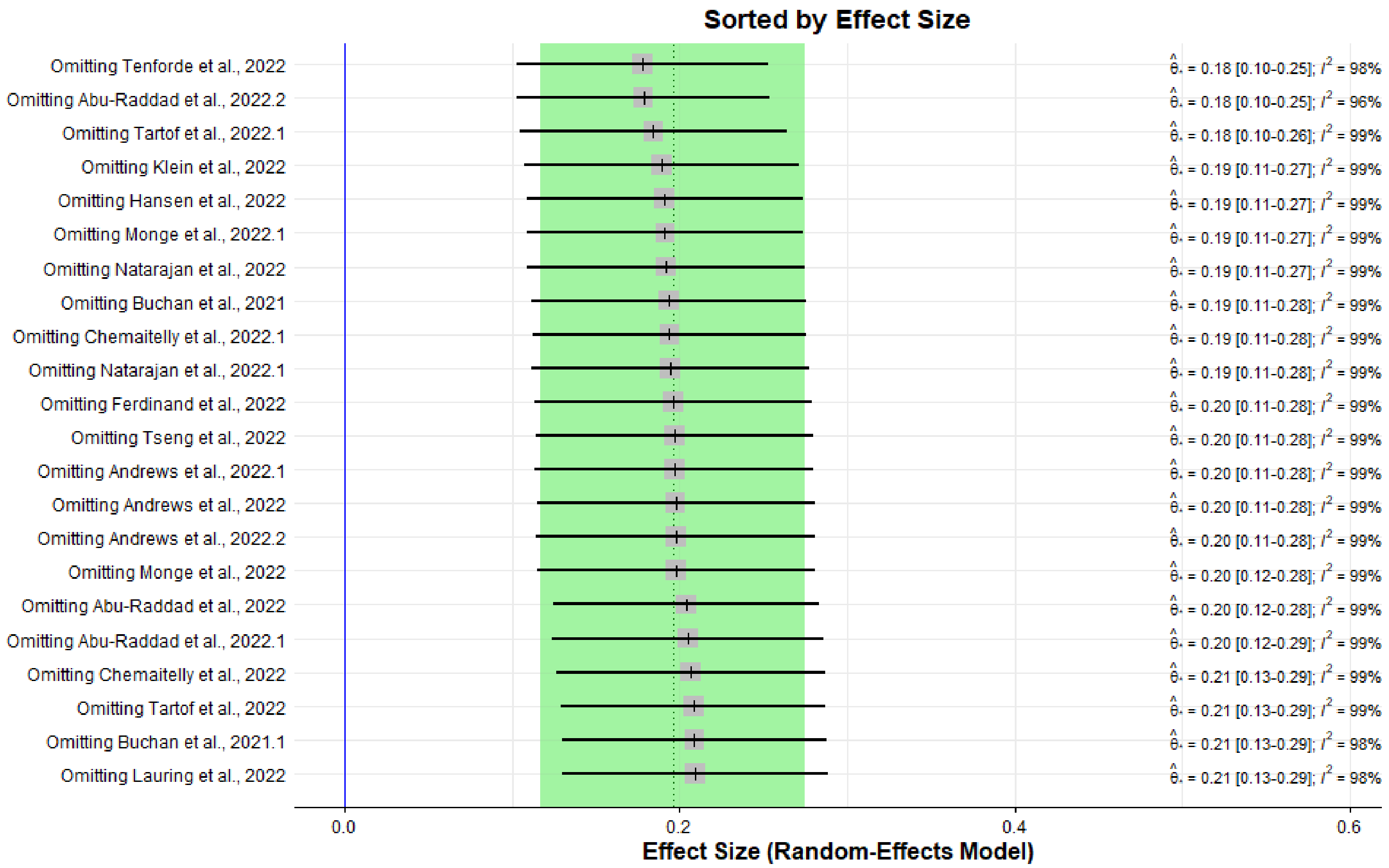

Figure S2. Sensitivity Analysis for Symptomatic Infection Endpoints of 0 to 3 Months Model

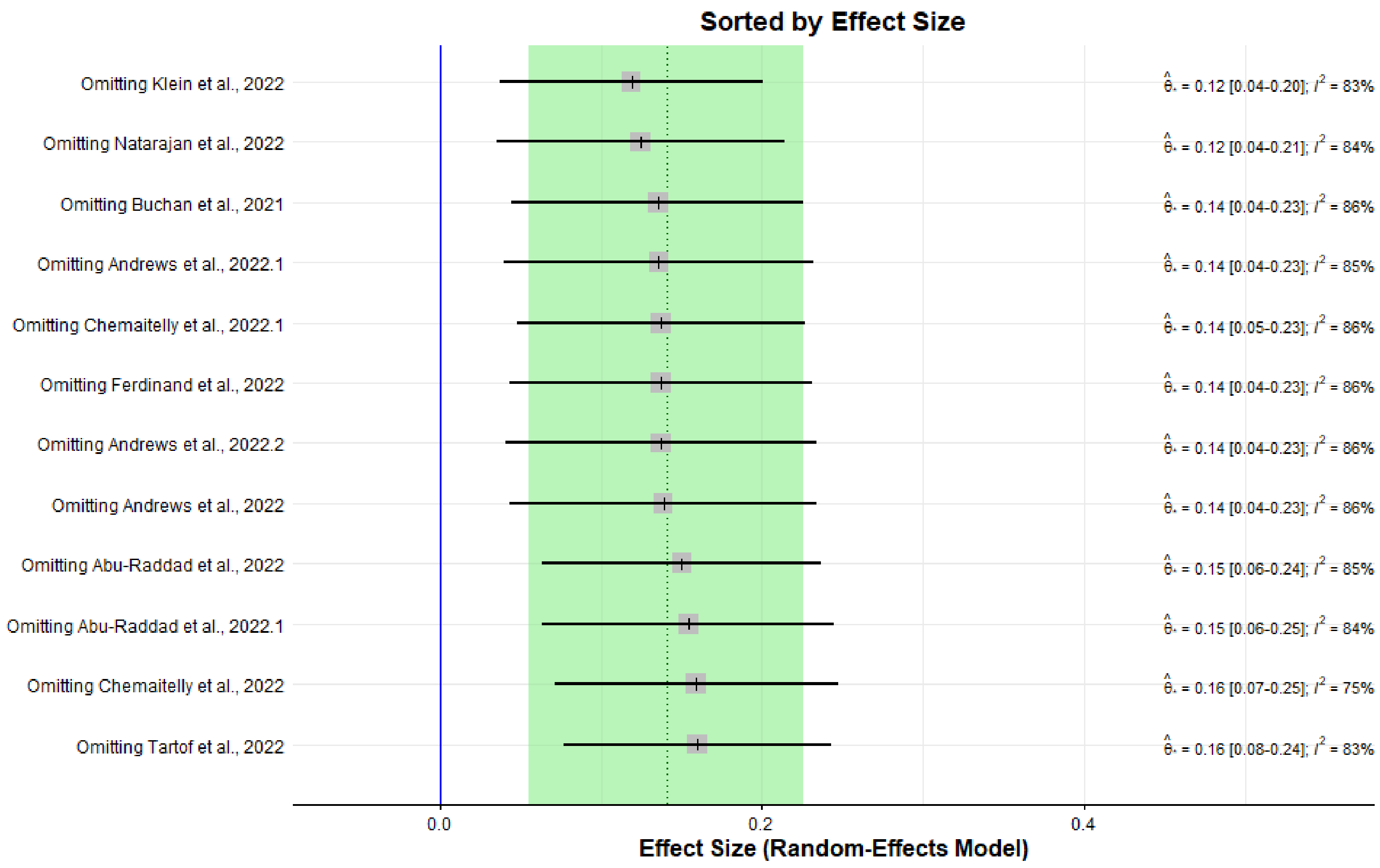

Figure S3. Sensitivity Analysis for Severe Infection Endpoints of 0 to 3 Months Model

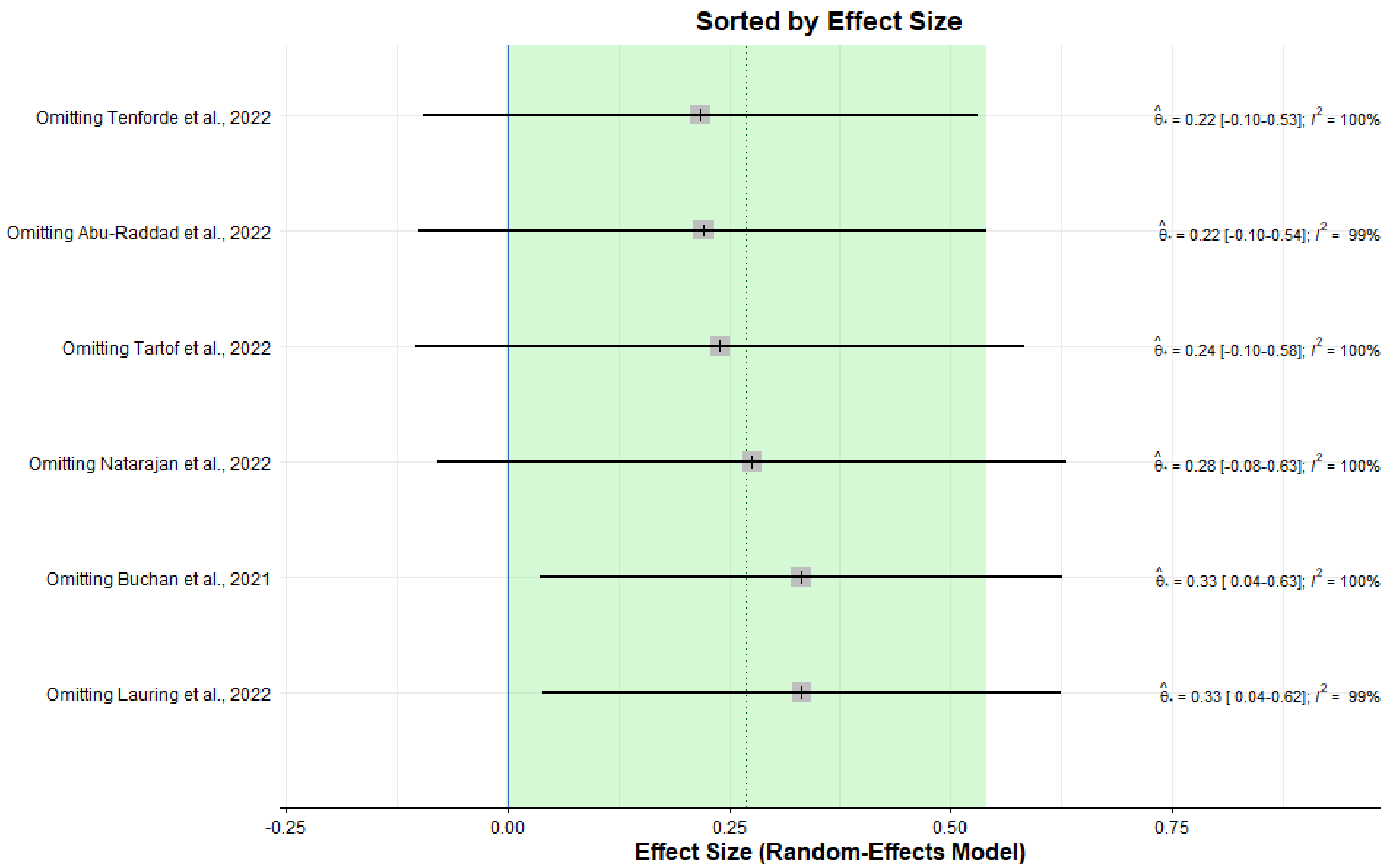

Figure S4. Sensitivity Analysis for Any Infection Endpoints of 0 to 3 Months or More Model

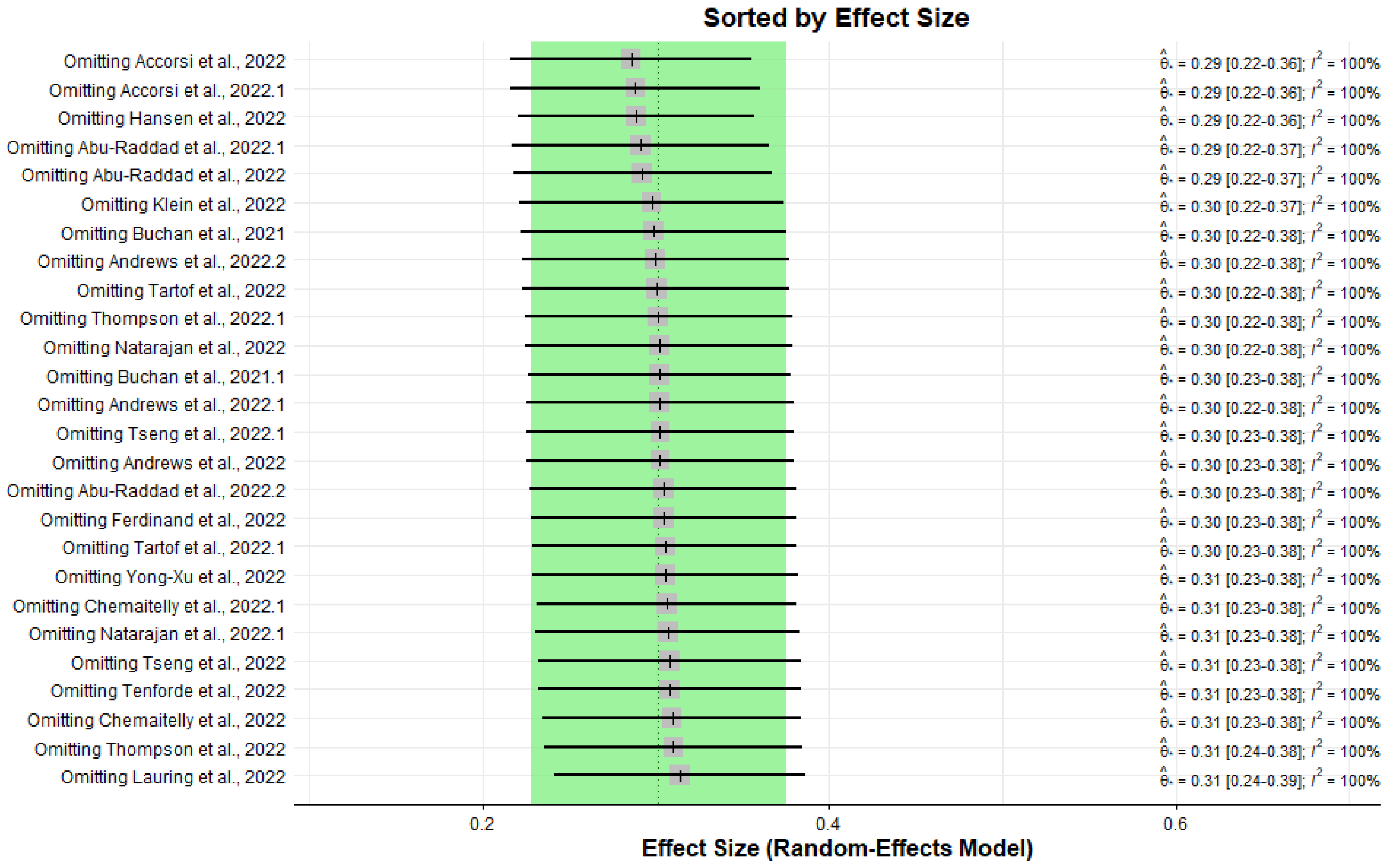

Figure S5. Sensitivity Analysis for Symptomatic Infection Endpoints of 0 to 3 Months or More Model

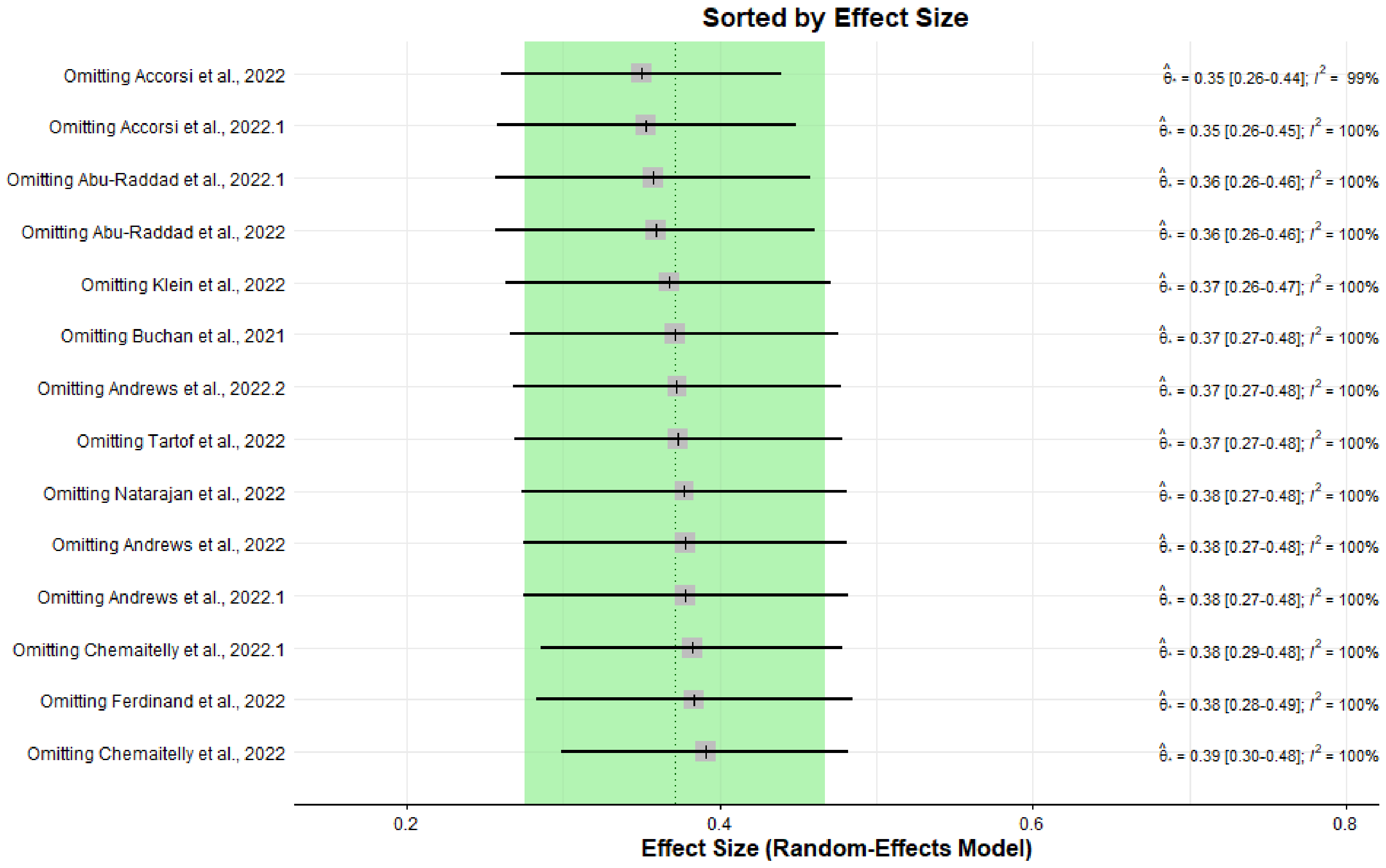

Figure S6. Sensitivity Analysis for Severe Infection Endpoints of 0 to 3 Months or More Model

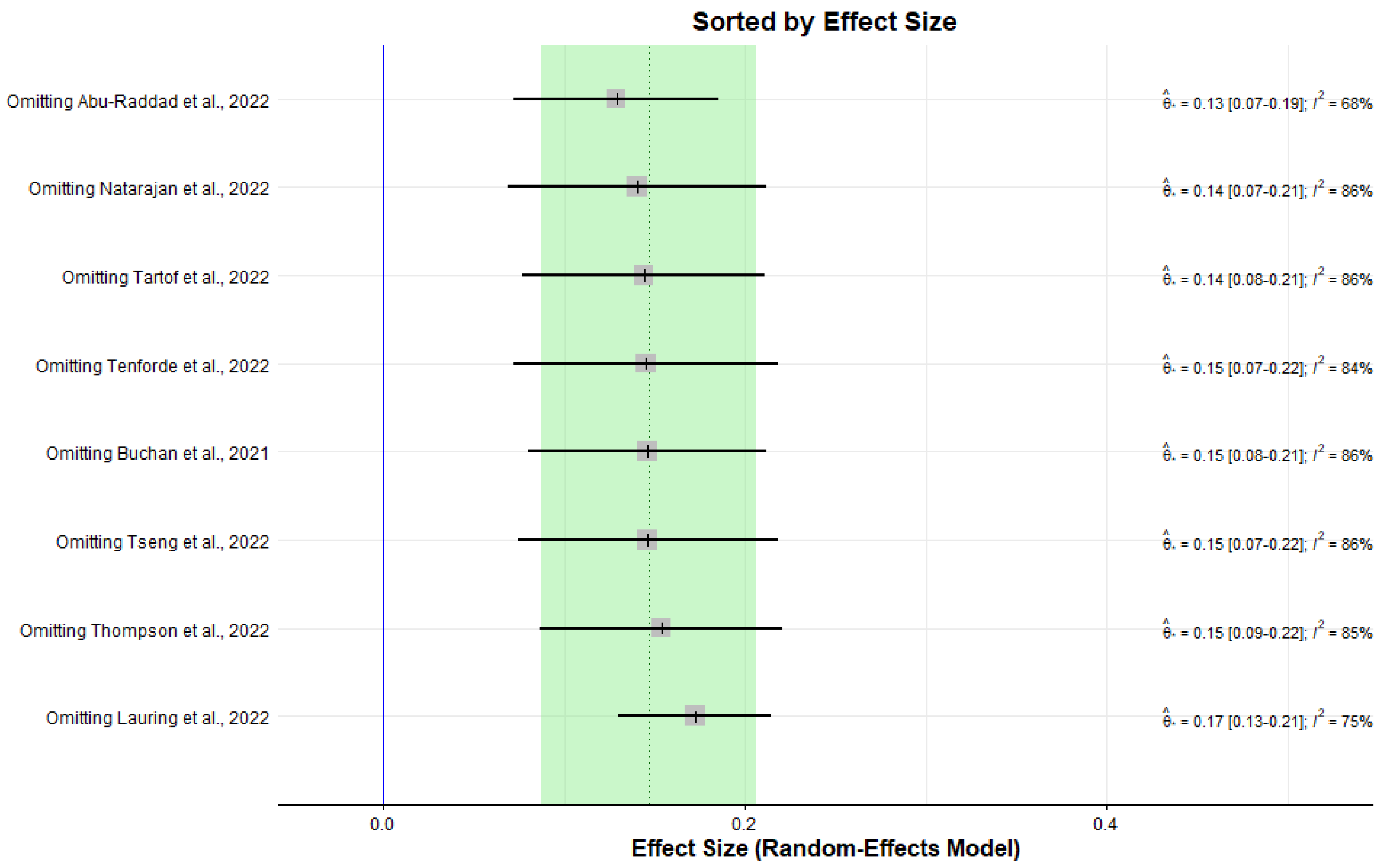

Supplement: Supplementary file 1 [file vaccines-10-02180-s001.zip › vaccines-1977691-supplementary.pdf]
